# Voice estimation in patients after reconstructive subtotal laryngectomy

**DOI:** 10.1186/1758-3284-3-46

**Published:** 2011-10-26

**Authors:** Bożena Wiskirska-Woźnica, Małgorzata Leszczyńska, Świdziński, Hanna Czerniejewska, Joanna Jackowska, Szyfter Witold

**Affiliations:** 1 Department of Phoniatrics and Audiology Poznań University of Medical Sciences, Poland; 2 Department of Otolaryngology Poznań University of Medical Sciences, Poland

## Abstract

**Background:**

Treatment of laryngeal cancers, may include surgery, radiotherapy, chemotherapy, or a combination. Total laryngectomy (TL) has been the standard surgical treatment. Partial laryngectomy procedures were performed, their advantage over TL is preservation of laryngeal functions.

**Methods:**

The investigation was carried out on a group of 20 patients (3 female and 17 male), who underwent surgery according the techniques mentioned above. The methods of investigation were based on perceptual voice estimation (GRBAS), videolaryngostroboscopy, acoustic voice analysis, aerodynamic measure maximum phonation time, voice self-assessment (VHI).

**Results and Conclusions:**

The perceptual voice estimation revealed a good phonation result in only 3 cases after using surgery with the Calearo method as well as the best results of MPT. The VHI reflected severe voice handicap in 2 patients (26 to 40 points). No statistically significant differences were observed between the values of the acoustic parameters in MDVP analysis after following operation -CHEP, Calearo, Sedlacek.

## Background

Treatment of laryngeal cancers, one of the most common types of head and neck cancer, may include surgery, radiotherapy, chemotherapy, or a combination. Optimal primary treatment depends on the experience and philosophy of the responsible surgeon and the infrastructure at the institution. For many years total laryngectomy (TL) has been the standard of surgical treatment for advanced stage of laryngeal carcinoma. From the surgical point of view the organ preservation strategy includes surgical procedures which, if possible, preserve physiological speech, swallowing and respiratory function.

**Figure 1:**
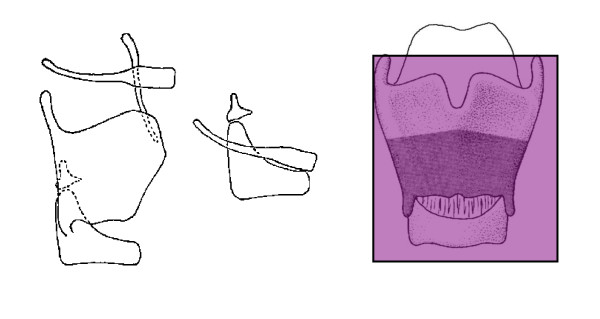
**Supracricoid laryngectomy with CHP**.

Supracricoid partial laryngectomy procedures i.e., those (SCPL) are conservative surgical techniques for the treatment of selected laryngeal carcinomas [1], is classified as T1-T4. SCPL includes cricohyoidopexy (CHP), which was reproposed by Labayle and Bismuth in 1972 [2] after the original studies of Serafini [3] on reconstructive laryngectomy, cricohyoidoepiglottopexy (CHEP) [or subtotal laryngectomy, according to Majer-Piquet], and tracheocricohyoidoepiglottopexy (TCHEP). The advantage of SCPL over TL is, that a permanent tracheostoma is not required since the main laryngeal functions of respiration, phonation and swallowing are preserved [4-6]. CHP is suitable for advanced supraglottic carcinoma involving the glottis, ventricle, or anterior commissure [6-8]. Typically it includes removing the whole thyroid cartilage, both the true and false cords, the ventricles, the epiglottis, and the paraglottic and preepiglottic region. At least one arytenoid cartilage must be spared to preserve phonation and sphincteric function. Reconstruction is accomplished by pulling together the hyoid bone and cricoid cartilage and then suturing them together [9]. Figure 1

**Figure 2:**
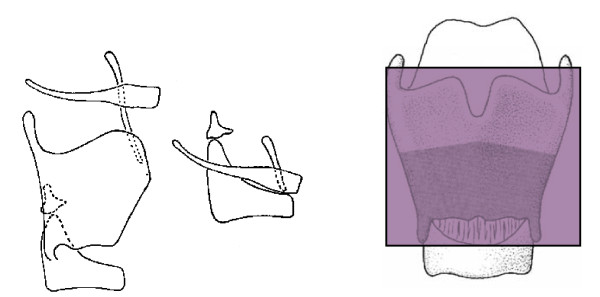
**Supracricoid laryngectomy with CHEP**.

CHEP involves resection of the infrahyoid part of the epiglottis and the preepiglottic. Reconstruction consists of suturing the hyoid bone, suprahyoid epiglottis and cricoid cartilage closely together [9]. Figure 2

The same structures as in CHEP are removed in TCHEP, the difference being only in the reconstruction which is performed by suturing the epiglottis to the first tracheal rings, since the anterior arch of the cricoid cartilage is resected. The surgical technique must preserve the recurrent laryngeal nerve, which runs between the cricoid carilage and the inferior horn of the thyroid cartilage, to allow the recovery of both phonation and sphincter functions [9].

**Figure 3:**
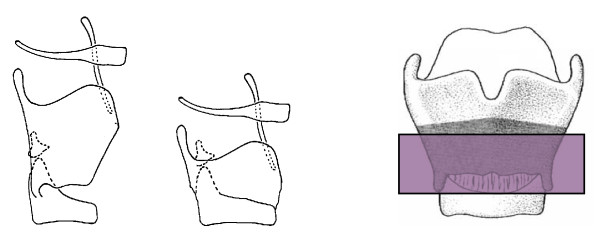
**Transglottic laryngectomy according to Calearo**.

The Calearo technique is a method of reconstructive laryngectomy which can be adopted in two types of surgical techniques used in the treatment of intrinsic laryngeal tumors. This methods in which one or both of the arytenoids are conserved, can be applied in cases of glottic neoplasms extending to both vocal folds with, sometimes, infiltration of the vocal process and glottic cancers where a simple cordectomy is not feasible [10]. Figure 3

**Figure 4:**
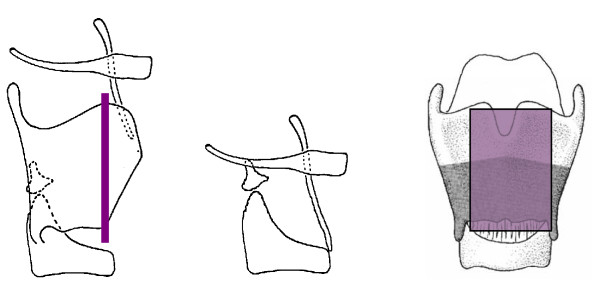
**Transglottic laryngectomy according to Sedlaček-Tucker**.

The Sedlacek-Tucker procedure is a modification of this operation. The lateral margins of the epiglottis with the aryepiglottic fold, are sutured to the arytenoid region of the cricoid rather than to a thyroid cartilage remnant, thereby forming a neo-arytenoid. The lateral margin of the epiglottis with the aryepiglottic fold is sutured to the cut edges of the false and true cords. Both margins of the epiglottis, together with the aryepiglottic folds, are lowered to the level of the glottis as much as possible to form a new pseudocord. A cartilage incision is made at the anterior aspect of the epiglottis, leaving its laryngeal surface of mucoperichondium intact. A new anterior commissure with a sharp angle is shaped by this maneuver [11]. Figure 4

Supraglottic laryngectomy is an accepted treatment for some patients with primary T2 stages, and some T3 cancers of the supraglottic region.

Since the aim of the ENT surgeon is to preserve main laryngeal functions: respiration, swallowing and phonation as much as it is possible was an attempt to evaluate this study and compare the long-term results of phonation in a group of patients who had undergone SCPL with either CHP, CHEP, the Calearo and Sedlacek procedures or supraglottic laryngectomy.

## Materials and methods

The investigation was carried out on a group of 20 patients aged 52-82 years (mean 66, 5), consisting of 3 female and 17 male patients, who underwent surgery due to laryngeal carcinoma: after SCPL in four cases (T3N0M0), Calearo in eleven (T2N0M0), Sedlacek in three (T2N0M0) and after supraglottic laryngectomy in two cases (T2N0M0). No additional radiotherapy was given.

The methods used in the investigation were based on (1) perceptual voice estimation on the GRBAS scale, (2) videolaryngostroboscopy of the neoglottis, (3) acoustic voice analysis by the Kay technique, (4) maximum phonation time(MPT) as a simple aerodynamic measure, and (5) voice self-assessment on the VHI scale.

The GRBAS scale was used for perceptual voice ratings by three persons, a phoniatrician, a laryngologist and a general practitioner during spontaneous conversation. The results are given as the arithmetic mean. The GRBAS scale consists of 5 parameters of the voice: G- grade of hoarseness, R - rough, B- breathy, A- asthenic, S- strained, scored on a four- point scale, where 0 is a normal voice, and I = light, 2 = moderate and 3 = severe change.

In videolaryngostroboscopy, performed using a rigid endoscope with the Wolf device and a CCD camera, the following variables was estimated: a) the vibratory characteristics of the neoglottis, b) the degree of arytenoid motion and c) anterior-posterior valving of the arytenoid/epiglottal/base of the tongue complex during various phonation efforts.

Acoustic voice analysis was performed using an IBM computer and specific software of the Kay Elemetrics CSL 4300 Model. The voice samples of each patient were recorded individually in a standardized way and always in the morning with a microphone positioned approximately 15 cm from the mouth, and slightly below the chin, to reduce airflow effects. Each patient was asked to estimate his daily vocal load.

The Multi Dimensional Voice Program (MDVP) was used to perform an objective voice evaluation with the following parameters:

**- **Amplitude Perturbation Quotient - APQ; %

- Amplitude Tremor Intensity Index - ARTI; %

- Degree of Sub-Harmonics - DSH; %

- Degree of Voiceless - DUV; %

- Degree of Voice Breaks -DVB; %

- Fo Tremor Intensity Index - FTRI; %

- Jitter Percent - Jitt; %

- Noise-to-Harmonic Ratio - NHR;

- Pitch Period Perturbation Quotient -- PPQ; %

- Relative Average Perturbation - RAP; %

- Smoothed Amplitude Perturbation Quotient - sAPQ; %

- Shimmer Percent - Shim; %

- Soft Phonation Index - SPI

- Smoothed Pitch Perturbation Quotient - sPPQ; %

-Peak Amplitude Variation - vAM; %

- Fundamental Frequency Variation - vFo; %

- Voice Turbulence Index - VTI.

Finally, each subject completed the Jacobsons Voice Handicap Index (VHI). This is a validated instrument designed to assess the patient's self-perceived emotional, physical a functional complaints, relative to their vocal dysfunction. The VHI score is based on a standard Likert analysis of subject responses to a estimation as a voice handicap.

## Results

The perceptual voice estimation (GRBAS scale) during spontaneous conversation revealed a good phonation result in only 3 cases after using surgery with the Calearo method. Theirs voice was slight hoarse, rough and strained. The voice of another 8 patients, after various types of partial laryngectomy, mostly after CHEP, was classified as severe, hoarse and rough, and even very weak in one case. The remaining 9 patients showed mild dysphonia on the GRBAS scale (Table 1).

**Table 1:** Parameters and rates used in the voice evaluation

Patient number	MPT (sec)	GRBAS scale	VHI score
1/CHEP	6	G2R2B1A1S2	41

2/Calero	5	G2R2B1A1S1	16

3/CHEP	8	G3R3B2A1S3	17

4/Supraglottic	7	G1R1B0A0S0	11

5/Calearo	8	G2R3B2A1S2	48

6/Calearo	4	G2R3B1A1S2	67

7/Calearo	18	G3R2B1A0S1	17

8/Sedlacek	6	G2R1B2A1S2	22

9/Calearo	5	G3R2B2A1S2	32

10/Sedlacek	17	G2R1B1A0S2	11

11/Supraglottic	9	G2R1B0A0S1	17

12/CHEP	2	G3R2B1A0S2	104

13/Calearo	21	G2R1B0A1S2	15

14/Calearo	7	G3R2B1A2S2	5

15/CHEP	14	G2R2B1A1S2	23

16/Calearo	6	G2R0B2A1S1	26

17/Calearo	5	G1R0B0A0S1	19

18/Calearo	8	G3R2B2A2S0	52

19/Sedlacek	4	G3R3B3A2S2	49

20/Calearo	14	G1R1B1A0S1	43

The maximum phonation time (MPT) in all patients varied from 2 to 21 s (mean 10 s). The best results of MPT were noticed after reconstructive surgery with the Calearo method (Table 1).

Voice self-assessment using the VHI scale reflected severe to mild degrees of voice handicap (respectively 5 to 25 points) in 10 patients, and 26 to 40 points in 2 patients (max. score 120, Jacobson scale results below 60 points- voice handicap). Only one patient who assessed his voice as almost normal, scored 104 points on the VHI scale. Comparing the median results of VHI to the types of partial laryngectomy, the worse score, 27, was obtained after the Sedlacek operation, 31 after surgery with the Calearo method and the best score, 46, was registered after CHEP (Table 1).

**Figure 5:**
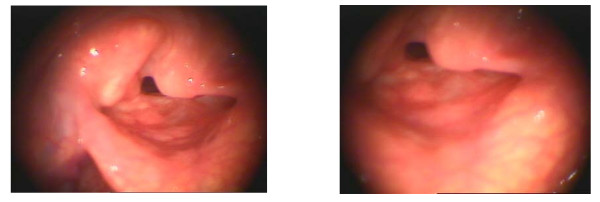
**Neoglottis - after Calearo method surgery**.

**Figure 6:**
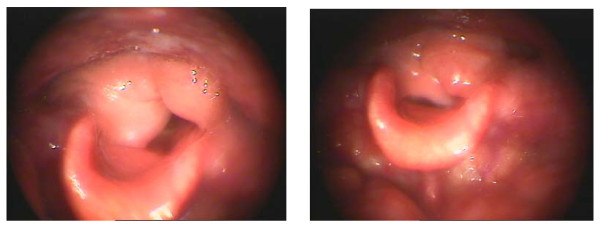
**Neoglottis - after CHEP**.

**Figure 7:**
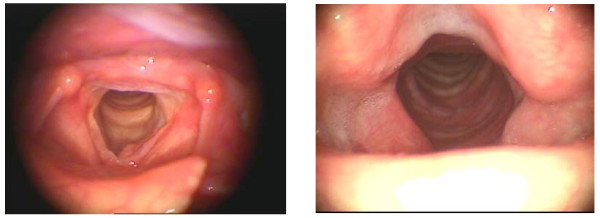
**Neoglottis - after laryngectomy by the Sedlacek method**.

The results of laryngostroboscopic findings regarding the shape of the neoglottis and phonation closure are shown in Table 2 and Figures 5, 6 and 7.

**Table 2:** Types of neoglottis in our group of patients after partial laryngectomy

Types of neoglottis and phonation closure	Number of cases
One vocal fold and epiglottis	7

Laryngeal surface of epiglottis, 2 arytenoid cartilages and 2 pseudo-folds	7

Laryngeal surface of epiglottis and 2 arytenoid cartilages	1

Scar after epiglittis and 2 aryepiglottic folds	2

Pseudo vocal folds	1

Pseudo- folds without arytenoid cartilages	2

**Figure 8:**
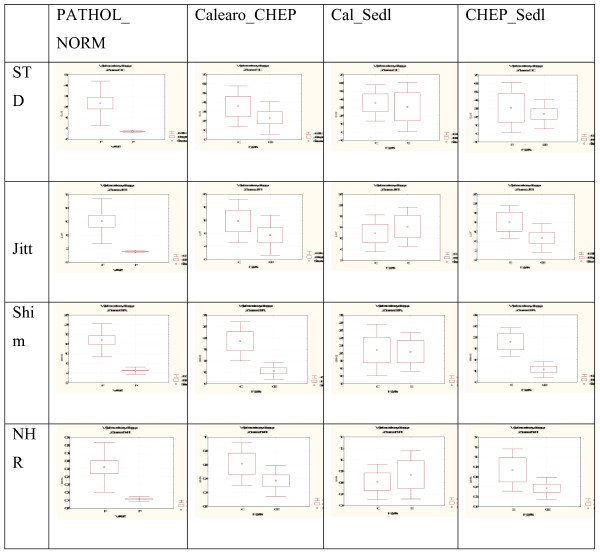
**Comparison of mean values of Jitt, Shim, NHR and standard deviation between groups: norm/patol., Cal/CHEP, CAL/Sedl., CHEP/Sedl**.

Due to the quality of recorded voice samples, acoustic analysis was performed only in 14 patients. The results using the MuliDimensional Voice Program (MDVP) are shown for all those investigated, with significant statistical differences (Mann-Whitney Test p < 0.05) only occuring for parameters describing amplitude perturbances (SHdb, Shimm, APQ). All our acoustic results are presented in Tables 3 and Figure 8.

**Table 3:** The relevance of differences between the acoustic results of the various types of operation in comparison to norms (Mann-Whitney test)

	CALEARO	CHEP	p	CHEP	SEDL	p	CALEARO	SEDL	p	Pathology	Norm	p
STD	7	4	0.5273	4	3	1	7	3	0.8571	14	61	**0.0000**

JITA	7	4	0.5273	4	3	0.1833	7	3	0.8571	14	61	**0.0000**

JITT	7	4	0.2303	4	3	0.1833	7	3	0.6286	14	61	**0.0000**

RAP	7	4	0.9273	4	3	0.1167	7	3	0.2286	14	61	**0.0000**

PPQ	7	4	0.2303	4	3	0.3833	7	3	0.6286	14	61	**0.0000**

SPPQ	7	4	0.3152	4	3	0.2667	7	3	0.6286	14	61	**0.0000**

VFO	7	4	0.6485	4	3	0.6667	7	3	0.2286	14	61	**0.0000**

SHDB	7	4	**0.0242**	4	3	**0.0333**	7	3	0.8571	14	61	**0.0000**

SHIM	7	4	**0.0242**	4	3	**0.0333**	7	3	0.8571	14	61	**0.0000**

APQ	7	4	0.0727	4	3	**0.0167**	7	3	0.6286	14	61	**0.0000**

SAPQ	7	4	0.2303	4	3	0.0667	7	3	1.1429	14	61	**0.0000**

VAM	7	4	0.6485	4	3	0.5167	7	3	0.8571	14	61	**0.0000**

NHR	7	4	0.3152	4	3	0.1167	7	3	0.8571	14	61	**0.0000**

VTI	7	4	0.6485	4	3	0.1833	7	3	0.4	14	61	**0.0000**

SPI	7	4	0.7879	4	3	0.1167	7	3	0.4	14	61	**0.0000**

DVB	7	4	0.1091	4	3	0.3833	7	3	0.1143	14	61	**0.0119**

DSH	7	4	0.1636	4	3	0.5167	7	3	0.2286	14	61	**0.0119**

DUV	7	4	0.3152	4	3	0.2667	7	3	0.4	14	61	**0.0000**

NVB	7	4	0.3152	4	3	0.3833	7	3	0.1143	14	61	**0.0119**

NSH	7	4	0.1636	4	3	0.6667	7	3	0.2286	14	61	**0.0119**

NUV	7	4	0.3152	4	3	0.3833	7	3	0.8571	14	61	**0.0000**

No statistically significant differences were observed between the values of the parameters in the comparison of acoustic parameters in MDVP analysis after three different types of operation -CHEP, Calearo, Sedlacek. However the absolute values of frequency parameters (Jitt, RAP, PPQ), which were estimated from a small voice sample, were the best after the Sedlacek procedure and the worst after the Calearo method. Analysis of fundamental frequency (SP, PQ, vFo) revealed the best results following Calearo and the worst after CHEP. Indicators of amplitude variations (Shim, APQ, sAPQ) and parameters describing noise component (NHR, VTI) were the best after Sedlacek and the worst after Calearo.

In summary, the best results in acoustic analysis, particularly of frequency and amplitude perturbation, were observed after Sedlacek, but, conversely, perceptive voice estimation and voice self-assessment were worst in this group of patients.

After the Calearo operation, where perceptualy the voice was the best, the results of acoustic analysis except fundamental frequency were definitely worse.

## Discussion

The voice characteristics of a group of 20 patients who had undergone SCPL were assessed using endoscopic, aerodynamic, perceptual, acoustic and self-assessment ratings.

The changes which occur in residual laryngeal anatomo-physiology following SCPL cause reasonable doubt that most parameters and the traditional methods used in vocal evaluation cannot from a physiological, physical and acoustic standpoint, be taken into consideration after this type of surgery [12].

Acoustic evaluation of the patient's post-operative voice requires objective and quantitative acoustic analysis to investigate the results hopefully and to upgrade the phonatory results of surgery. Modern acoustic digital analysis of the vocal sound (noise, harmonics, frequency and intensity short-term variations, etc.) can still be used to obtain various measures of vocal quality as well as to provide information of the " neoglottis" regarding the functional results. These methods allow easier and less subjective comparisons of acoustic functional results in surveys [13].

Acoustic features of the voice of patients submitted to SCPL are related to both the remaning anatomical structures and to the functional abilities of the residual phonatory system. Hence, the vibrational pattern of the " neoglottis" generally appears rather unstable and not-always-periodic, because of the altered anatomical characteristics of the various vibrating structures. i.e.,[14,15]:

1. arytenoid mucosa with no structure which can be modulated lying below;

2. tongue base/pharynx/epiglottis;

3. a T-shaped neoglottis (with 2 arytenoids);

4. an upside-down L-shaped;

5. closure modes (sagittal, front, mixed);

6. incomplete closure.

Some authors have compared the vocal characteristics after SCPL with CHP or CHEP, from both quantitative and qualitative points of view.

Traissac et al. [16] analysed 122 cases, of which 97 were following CHEP and 25 CHP. A good voice was achieved in 25% of patients treated with CHEP and in 17% of those treated with CHP. The voice was not restored in those patients treated according to SCPL-CHEP and in 60% of those who underwent SCPL-CHP. Finally, in 17% and 23% of their patients treated, respectively, with CHEP and CHP, an understandable voice was noted. No big differences between results, obtained shortly after surgery and those after a rehabilitation program, were noted which is in contrast to the experience of other authors. In fact, to this end, Minni et al. [17] reported that the intensity of vocal production become more and more dynamic, continuous and regular after surgery and, furthermore, in the opinion of these authors, early treatment of speech defects, by guaranteeing fast recovery of laryngeal physiological function, allows a more rapid return to social life of those patients submitted to SCPL.

In 103 patients submitted to CHP, Labayle and Dahan [18] observed improvements occurring over months. This improvement was often related to the decision of the patient whether to undertake rehabilitation, or not. The improvements, following rehabilitation, were impressive.

Piquet et al. [19] studied 117 patients, of whom 71 submitted to CHP and 46 CHEP. Vocal quality was good in 80% of cases, being better and with a sound production intensity higher than prior to treatment and with a low-pitched timbre. In the 20%, the restored voice qualified as poor, even after rehabilitation.

Pech at al., [20] evaluating phonatory function in a group of 49 patients, 17 of whom were following CHEP and 32 following CHP, observed good recovery of the voice in all the CHEP-treated patients, while in the 32 CHP-treated patients voice quality was poor. Nevertheless, as the authors stress, the worst voice in these patients is always better than the oesophageal voice, certainly in the absence of a tracheostoma.

Vigneau et al. [21] compared the functional results of 64 patients submitted to SCPL from 1975 to 1985, of whom 52 underwent CHEP and 8 CHP, with 4 patients who had undergone total laryngectomy due to a previous resection which was considered oncologically insufficient. According to the rehabilitation protocol, the beginning of orthoepical retraining was programmed to begin 10 days after surgery, together with external massage. A good, perfectly understandable, and satisfactory voice was achieved in 69% of CHEP speakers and in 60% of CHP speakers. In 21% and 22%, of CHEP speakers and CHP speakers, respectively the voice was slightly voiced, of low intensity but understandable and considered satisfactory by the patients. The remaining 10% of the CHEP speakers and 11% of the CHP speakers had a residual voice which was hardly understandable and, in general, of poor quality.

Prades and Martin [22] observed 19 patients submitted to CHP and referred to the quality of voice as always being good. As far as concerns this result, an essential role was played by the mobility of the arytenoids and by the fact that, despite a reduced anteroposterior diameter, the width and height of the laryngeal canal were preserved, thus allowing better vibration of the structures when the air column passes.

Guerrier et al. [23] studied functional ability in 58 patients, all affected by laryngeal glottic carcinoma, who had been submitted to CHEP. After a minimum observation of at least 4 months, the results demonstrated good phonatory recovery in all patients. Factors influencing voice quality, besides preservation of the arytenoids, are motivation, but above all, the patient's educational ability allowing him/her to gain the greatest profit from the various orthoepical rehabilitation manoeuvres.

Ferri And Bottazzi [24], in 21 patients with SCPL, observed a good recovery of phonatory quality in 5 (23%); sufficient in 10 (47%) and poor in 6 (30%).

Marandas et al. [25] in a survey of 57 patients submitted o CHP, observed poor phonatory results in 16 patients (28%) and good in 41 (72%).

Prades et al. [26] analysed 2 patients who underwent CHEP, concluding, from the results, that phonation is basically a source of complaint among the patients, as well as a strain due to closure of the glottis. These two functions of the neolarynx are, as a rule, of poor quality. Moreover, there is little difference between the results of the various surgical techniques, and even these are very difficult to define.

Pastore at al. [27] submitted the recorded phrases of 14 patients, following reconstructive subtotal laryngectomy treatment, to the attention of trained listeners. This study proved that vocal quality after surgery, although little voiced, permits an understandable and socially acceptable level of communication.

Laccourreye et al. [28,29] investigated the functional results in 104 patients following SCPL (68CHP and 38CHEP). All their patients showed good recovery of phonatory function thanks to the degree of tissue preservation, which is, as already pointed out, the main feature of this treatment.

De Vincentiis et al. [30] submitted 153 subjects to acoustic analysis, 83 underwent CHP, and 70 CHEP. All were submitted to perceptive analysis of the voice, vocal extension by means of the Fo indicator of the stroboscope and maximum intensity evaluation using a phonometer. The study showed better vocal recovery after CHEP, but the most important information was related to the maximum phonation intensity which provided most patients with asocially acceptable and useful phonation.

Genovese et al. [31] reported that, although the new voice achieved through SCPL is less sonorous, it is perfectly understandable, socially acceptable, speech.

Moreover, there are other studies in which the phonatory function of patients submitted to SCPL was evaluated by semi-objective methods.

Minni et al. [32] analysed 149 patients submitted to SCPL. Their functional evaluation included phonation and return to social life, as well as an analysis of vocal quality, by means of spectography. Although, in all cases, phonatory recovery was considered sufficient, typical phonatory features were observed in patients treated with this procedure, resulting in: a slowing of the speaking rate, lowering of the fundamental frequency and a constant increase in the noise component compared to that of the fundamental signal. The authors stressed the importance of post-operative rehabilitation which implies the reduction in noise in favour of the harmonics. Bonnet et al. [33] analysed the main physical features of the voice produced by the neoglottis, in 43 CHP speakers and in 68 CHEP speakers. In all the patients the voice was considered sufficient with a maximum intensity of 50-90 dB and a variable fundamental of approximately 120 Hz.

Laccourreye et al. [34], also revealed, by means of acoustic measurement, a considerable reduction in MPT, Speech Rate (number of words per minute), Phrase Grouping (number of words per breath), as well as an excessive Fo variability, a statistically significant increase in jitter, shimmer and NHR.

According to Jemmi et al. [35], SCPL associated with CHP, makes speech continuity possible. Such subjects have a mixed vocal output (periodic components together with noise) although, overall, the voice produced has the fundamental requirements for intelligibility (i.e., intensity, pitch, harmonic structure, emission time) and may thus be considered valid for interpersonal verbal communication.

In fact, Dejonkere et al. [36], proposing the ELS protocol for the functional assessment of the voice in "common dysphonia", define "substitution voices" as those in which the signal does not originate from the two vocal folds. They suggest re-addressing a specific protocol after acoustic analysis. In fact, the most important acoustic finding is the high variability of the fundamental frequency (when a nearly - periodic signal is generated!) caused by radical anatomical changes after SCPL.

Yuceturk [37] performed a multidementional assessment of voice and speech after supracricoid laryngectomy with CHP; The study evaluated vocal function in patients with SCPL compared with that in normal subjects. The acoustic parameters were found to be significantly different from those of normal subjects. The values of perceptual scores were within approximately 50% of the normal range. The number of arytenoids spared did not affect either acoustic or perceptual measurements. A rough, breathy, unpleasant, but intelligible and acceptable, voice could be obtained following SCPL with CHP.

According to Bron et al. [38], the restoration of laryngeal function after SCPL with CHEP is satisfactory. Although most of the patients seem to recover normal swallowing function, severe voice alterations appear to be inevitable.

Moerman et al. [39], have suggested that "substitution voicing" cannot be evaluated accurately by the GIRBAS perceptual rating scale, and therefore a valid alternative is needed.

In our patients treated by supracricoid partial laryngectomy(SCPL), with the Calearo or Sedlacek techniques the best results were achieved with Calearo. From the surgical point of view this organ preservation strategy includes surgical procedures which preserve physiological speech, swallowing and respiratory function. The Calearo procedure enables the creation of a neoglottis whose function is the most similar to the physiological as the phonatory closure of the neoglottis is generally produced by the ventricular folds. This fact is also confirmed by acoustic analysis. After other types of surgery the results of the acoustic analysis were not so favourable but, in comparison to those following total laryngectomy where the communication is based on esophageal speech, the results are satisfactory. Also the patient's self-perceived emotional, physical a functional effects, relative to their vocal dysfunction, are much better than in patients after total laryngectomy.

Bron et al. [40] mentioned, that due to unstable and loose neoglottic closure after CHEP the maximum phonation time decreased. He pointed also that the chance to get good voice quality is intensive voice therapy by improving its stability and intensity. Comparing to his findings, in our patients the acoustic voice analysis showed also high level of parameters describing voice instability.

Tolga Kandogan, Aylin Sanal [41]. They analysed quality of life, functional outcomes and voice problems facing early cancer patients treated with the surgical techniques such as laryngofissure cordectomy, fronto-lateral laryngectomy, cricohyoidopexi. They established that Cricohyoidopexy group has given the lowest scores but the cordectomy has given the highest ones in three survey questions representing the quality of life, performances and new voice. The difference between the VHI and VHI-functional, VHI-physical, VHI-emotional scores in three patient groups were not statistically different. All of the patients evaluated that their new voices had similar functional, physical and emotional impact on their life.

In all of the patient groups, the quality of voice was found to be sufficient to hold a normal individual conversation. However, the voice was defined by the patients as hoarse and dull.

Mark et al. [42]. Authors performed local control and 5-years survival rates, which were similar for patients undergoing total laryngectomy and supracricoid laryngectomy. All patients demonstrated intelligible voice and effective swallowing function postoperatively, supporting supracricoid laryngectomy.

Previous researches have used acoustic analyses to characterize objectively the degree of persistent dysphonia exhibited by SCL patients. Jitter and shimmer levels were shown to be abnormally elevated as long as 18 months postoperatively, but measure of speech intelligibility, prosodic sufficiency, and number of words uttered per minute demonstrated that patients achieved near-normal performance on these parameters.

The physical effects of their voice impediments were rated as moderate, suggesting difficulties with voice strain and excessive physical effort required to produce intelligible voice.

In the present investigation, supracricoid laryngectomees demonstrated functional voice, speech, and swallowing abilities, although to varying degrees. Neoglottal incompetence resulted in a breathy-hoarse voice quality as graded by blinded expert listeners. All patients were rated to have highly intelligible speech.

Cagatay Oysu et al. [43]. Authors compared functional and oncological outcomes of cricohyoidoepiglottopexy (CHEP) and near-total laryngectomy with epiglottic reconstruction (NTLER) techniques in early glottic carcinoma.

Fundamental frequency, maximum phonation time, and maximum phonation intensity measurements were not significantly different in the 2 groups. There was also no significant difference in mean Voice Handicap Index score. According to the GBRAS scale, overall voice quality was moderately altered in both groups.

## Conclusion

The beneficial effects of conservative laryngeal surgery in respect to voice quality were estimated from perceptual estimation of neoglottis function from laryngostroboscopic examination, acoustic voice analysis and the patients voice self assessment. The best neoglottis function was achieved after partial laryngectomy by the Calearo method. Despite the less than perfect phonation achieved by this organ preservation strategy in comparison with total laryngectomy, the almost physiological levels of phonation are a factor of considerable important to the patients.

## Competing interests

The authors declare that they have no competing interests.

## Authors' contributions

BWW carried out examination of the neoglottis and interpretation of the data, coordinated and helped to draft manuscript. ML participated in the design of this study and sequence alignment, and helped to draft manuscript. PS performed the acoustic voice analysis and the statistical analysis. HC participated in phoniatric estimation and draft manuscript. JJ participated in the sequence alignment and phoniatric evaluation. All authors read and approved the final manuscript.
